# Unique health care utilization patterns in a homeless population in Ghent

**DOI:** 10.1186/1472-6963-10-242

**Published:** 2010-08-19

**Authors:** Evelyn Verlinde, Tine Verdée, Mieke Van de Walle, Bruno Art, Jan De Maeseneer, Sara Willems

**Affiliations:** 1 Department of General Practice and Primary Healthcare, Ghent University, Belgium

## Abstract

**Background:**

Existing studies concerning the health care use of homeless people describe higher utilisation rates for hospital-based care and emergency care, and lower rates for primary care by homeless people compared to the general population. Homeless people are importantly hindered and/or steered in their health care use by barriers directly related to the organisation of care. Our goal is to describe the accessibility of primary health care services, secondary care and emergency care for homeless people living in an area with a universal primary health care system and active guidance towards this unique system.

**Methods:**

Observational, cross-sectional study design. Data from the Belgian National health survey were merged with comparable data collected by means of a face-to-face interview from homeless people in Ghent. 122 homeless people who made use of homeless centres and shelters in Ghent were interviewed using a reduced version of the Belgian National Health survey over a period of 5 months. 2-dimensional crosstabs were built in order to study the bivariate relationship between health care use (primary health care, secondary and emergency care) and being homeless. To determine the independent association, a logistic model was constructed adjusting for age and sex.

**Results and Discussion:**

Homeless people have a higher likelihood to consult a GP than the non-homeless people in Ghent, even after adjusting for age and sex. The same trend is demonstrated for secondary and emergency care.

**Conclusions:**

Homeless people in Ghent do find the way to primary health care and make use of it. It seems that the universal primary health care system in Ghent with an active guidance by social workers contributes to easier GP access.

## Background

Homelessness is worldwide an important societal issue as it can be considered as unacceptable and unfair. In the USA, each year over three million people experience homelessness of which 1,3 million children [1]. In England 99.5000 households were officially recognised as newly homeless in 2007 [2]. In the Flemish region each year 12.000 people get support in residential care for homeless persons [3].

Compared to a decade ago, the homeless population today is younger (between 30 and 50 years) with a growing number of women and children. This trend continues both in the USA and in Europe [4-7]. The way people become homeless can be attributed to a number of factors such as a disruptive family environment characterized by extreme poverty, marital discord, addiction, financial problems (no job, no money) and mental health problems [7-10].

### Homelessness and health

Homeless people face many challenges. They lack the access to basic human needs such as shelter, clothing and healthy food, and have a disproportionally higher burden of disease than the average population [11]. They suffer from a wide range of medical problems, acute as well as chronic, e.g. tuberculosis, [8,12-14] hepatitis [7,14], HIV [4,7,14-16], influenza [13] and skin and soft tissue infections [8,13,15,16]. On top of the physical diseases, homeless people are more frequently caught in the downward spiral of having mental health problems or substance abuse: suffering from disorders such as depression, bipolar disorder or schizophrenia makes it even more difficult to find a job, stable housing or medical care [4,10,12,15,17]. These higher morbidity rates within the homeless population are also reflected in raised mortality rates [4,7,9,15]: studies on the European and US situation report life expectancies between 41 and 47 years [4-6,18,19].

### Homeless and health care utilisation

The literature concerning the health care utilisation of homeless people is scarce and almost exclusively reporting on the situation in the UK and the US. These studies show low utilisation rates of primary care among homeless people, with emergency care often being the only form of health care use [6,7,14,16,20-23]. As a result homeless people often consult with severe diseases and complications that could be prevented through earlier contact with a health care provider [22].

This particular pattern in health care use by homeless people is largely determined by the way the health care system is organised [7,20-23]. In the USA, the health care system relies almost entirely on the private sector for both the financing [24] and the delivery of the health care. Most health care facilities are privately owned and operated. Some vulnerable groups are covered by social insurance programs such as Medicare, Medicaid and the State Children's Health Insurance Program. Even though these programs contribute to a more accessible health care for those groups, they are not able to provide access to all types of medical care nor do they cover all groups of vulnerable patients [25]. Unsurprisingly, US based studies reporting on barriers in healthcare by homeless people describe several financial barriers to health care because of the lack of insurance (high insurance fees, refusing homeless patients because of missing identification cards,...), high costs of medication, competing priorities (finding housing or employment is more important than addressing health problems) [6,16,20]. Literature from the UK shows a slightly different picture, reflecting the particularity of the UK health care system. The UK has a universal health care system free at the point of service if people are registered with a GP [26]. It is in fact this registration procedure which forms an important barrier to primary health care for homeless people in the UK: they often don't know they have to register or are scared off by the complexity of the registration procedure, GPs are reluctant to accept them on their list,... [7,14,21,23,24].

Although the UK provides a health service for all, marginal groups, such as homeless people, are poorly served and sometimes excluded. Since 1997 the NHS has focused on improved primary health care services for these special needs groups [27]. Specialised general practices that register only homeless people have become more common [28]. But despite the installation of this selective health care system, parallel to the universal system, the majority of the homeless is not aware of its existence and use emergency care as a substitute for primary care [29].

Information on the health care use of homeless people in other countries and the influence of health system characteristics seems to lack.

Furthermore, literature also reports on a wide range of factors and mechanisms not related to the organisation of the health care system contributing to the particular health care use patterns of homeless patients. The majority of these studies emphasize the importance of the attitude of health care providers towards homeless patients. Care is often postponed because homeless people feel labelled, stigmatized or considered as "problem patients"[11,21].

In the region of Ghent (Belgium) the care for homeless people is organised in a unique way. In general, Belgium has a universal health care system with direct access to any general practitioner or specialist, without gate-keeping nor a patient-list. There is a fee-for-service system with about 30% co-payment for primary health care and 40% for specialist care. The lower socio-economic groups have a reduced co-payment of 8%. This co-payment is limited each year by an income-based threshold: the maximum bill [30]. Since 1982, the legal possibility to work in a capitation system in primary health care has been put in practice. In the area of Ghent (a midsized city in Belgium with 225 000 inhabitants) the 19^th ^century belt of deprived areas is to a large extent covered by Community Health Centres that provide interdisciplinary comprehensive primary health care using a capitation payment system. They adopt a universal approach in which all people residing in the neighbourhood can access the services of the centre. Less privileged residents such as the homeless staying in the area (e.g. frequenting the night shelters, living in squats, ...) are actively guided towards the community health centres by social welfare services. More specifically, there are formal agreements between the shelters for homeless people and the Community Health Centres in Ghent; in case people staying in the shelter need medical care, the nearby Community Health Centre is warned and (in most cases) a doctor comes to the shelter to see the patient. In rare occasions, the patient consults the doctor in the Community Health Centre (e.g. when he/she has been there before and he/she is able to walk to the Centre). Also for the payment of the costs, arrangements are made: all costs are directly refunded by the shelter or by the patient's Sickness Fund. When the patient is not insured, the Community Health Centres tend to ask no fee. Most homeless people living in the streets are usually known by the social workers working in the neighbourhood such as the outreachers and street corner workers. In case a homeless person needs medical care, the social workers refer him/her (or go with them) to one of the shelters (and usually do a follow-up whether the person did go to the shelter) or takes him/her directly to a doctor. There is no formal agreement between the street corner workers and the Community Health Workers, however the strong informal networks between them and the low financial and other thresholds of the Community Health Centres, makes that also the street corner workers usually lead the homeless people towards the Community Health Centres.

In one part of the area where there is no community health centre, people are guided towards a large group practice with a comparable way of working to the community health centres, except for the payment system which is fee-for-service based without co-payment for the patient.

Seen the particular organisation of the health care use in Ghent, we want to describe the accessibility of primary health care services, secondary care and emergency care for homeless people living in an area with a universal primary health care system and active guidance towards the system.

## Methods

The National Health Survey is a rich source of information when studying health and health care use. This cross-sectional survey is used to collect data from a representative sample of the non-institutionalised population residing in Belgium. For the 2004 survey, 12.945 Belgians were interviewed using both a face-to-face survey and a self-registering survey. Sampling has been based on a combination of stratification, multistage sampling and clustering. Stratification was done at the regional level and the level of the provinces. The number of selected individuals per province was determined by the proportions of the Belgians living in that province. In order to keep the fieldwork manageable, the number of interviews in each municipality should at least be 50. Per stratum, households were selected by randomly selecting so called 'reference persons' from the State Register. In every selected household, maximum four persons were questioned: the 'reference person', his/her partner and one or two other household members. Two hundred and thirty five inhabitants of Ghent were questioned. Finally, the data were anonymized by a trusted third party (the National Institute for Statistics) and made available for external researchers. For this study a variable was add to the dataset, indicating whether the participant is an inhabitant of Ghent.

However, homeless people are not included in the study. Therefore additional data from homeless people in Ghent was collected using a face-to-face survey, according to an adapted form of the National Health Survey protocol and using a reduced version of the NSH questionnaires.

The final database, used for this paper, contains data from 235 inhabitants with a fixed address in Ghent and data from 122 homeless people staying in a shelter in Ghent.

### Participants and sample

For this study the study population was restricted to all the homeless people making use of the homeless centres and shelters in Ghent. This choice was partly taken based on pragmatic reasons (homeless centres and shelters are the places where one can reach the homeless easily and with a good level of safety for the interviewers).

The exact total number of homeless people visiting shelters and homeless centres is not available however social workers estimated this total number at the time of the data collection on 250.

All the homeless people who made use of homeless centres and shelters in Ghent during the inclusion period of five months were invited to participate in the study by the researchers or the organisation's personnel (in most cases the social worker). Hereby no distinction was made between people occasionally sleeping in the shelter or people semi-permanently sleeping in the shelter.

Inclusion criteria for the study were: at the moment of the survey staying in an emergency or temporary accommodation in Ghent (shelter, homeless centre,...) (1), being homeless according to the definition of the Health Council (2), being aged between 18 and 65 years (3), and giving oral and written consent (4).

People who were noticeably intoxicated at the moment of the interview or were aggressive towards the interviewers were not included in the study as were those visitors who did not speak Dutch, French or English, or refugees. All participants gave written consent.

This study was approved by the Ethics Committee of Ghent University Hospital (project EC UZG 2006/060).

### Data collection

Data were collected by means of a face-to-face survey being a reduced version of the Belgian National Health Survey (questions on the gender, age and nationality of the respondent, his/her socio-economic status, health status and smoking, and health care use).

The following variables were used to study the possible confounding influence of other variables. *Socio-economic status*, based on the highest qualification the responded acquired and was categorised in 4 categories: no education/primary school, first 3 years of vocational school, vocational school completed (6 or 7 years), higher education/university. *Health Status *was measured by means of subjective health status (How is your health in general?). *Smoking status *was measured by 'Are you a current smoker'. The *utilisation of health care *was studied in terms of the likelihood to consult a GP, secondary care and emergency care and defined in terms of whether or not the individual consulted a general practitioner (GP), secondary care or an emergency department (ED) during the last two months.

Administrating the survey took place in a separate room of the organisation to respect the participant's privacy. Interviews lasted between 30 and 60 mins.

### Data analyses

The data were collected in the context of a larger study on the social gradient in health care use. For this paper, a selection of the database was used. More specifically the data from the homeless people were compared to the data from the 18 to 65 year old inhabitants of Ghent available in the most recent National Health Survey data base (2004). None of the latter were homeless. Data were analysed using SPSS version 16. Initially, 2-dimensional crosstabs with χ^²^-statistic were built in order to study the bivariate relationship between the homeless and non-homeless respondents: controlling for sex, age, education, nationality, subjective health, smoking behaviour and health care use. Secondly, 2-dimensional crosstabs with χ^²^-statistic were built in order to study the bivariate relationship between the health care use of the homeless and possible influencing variables such as sex, age, education, nationality, subjective health, smoking behaviour and type of practice. To determine the independent association between health care use and being homeless, a logistic regression model was constructed adjusting for age and sex. An odds ratio and 95% CI for contact with GP and contact with the ED in past two months was determined for each variable used in the model. The enterwise method was used to select the final model. The level of significance was tested by Wald tests, and P < 0,05 was set as being the level of statistical significance.

## Results

Table 1 describes the homeless respondents compared to the non-homeless respondents. It demonstrates that the homeless respondents are significantly younger (p < 0,001) and lower educated (p < 0,001) than the general population. Also the proportion of males (p < 0,001) and of people with a nationality other than Belgian (p = 0,002) is significantly higher in the homeless population. Forty percent of the homeless respondents report to be in fair, bad or very bad health, compared to 34 percent in the general population (p < 0,001) and the smoking rate is almost 3 times as high than in the general population (p < 0,001).

**Table 1:** Homeless people versus non-homeless people: descriptive and univariable results

	Homeless	Not homeless	
	**n (%)**	**n (%)**	P*

**Sex**	121	253	**< 0,001**
**Male**	101 (83,5)	124 (49,0)	
**Female**	20 (16,5)	129 (51,0)	
**Age**	121	253	**< 0,001**
**18-29**	42 (34,7)	71 (28,1)	
**30-39**	31 (25,6)	51 (20,2)	
**40-49**	31 (25,6)	56 (22,1)	
**50-59**	15 (12,4)	47(18,6)	
**60-65**	2 (1,7)	28 (11,1)	
**Educational level**	121	223	**< 0,001**
**No education/primary school** **lower education**	17 (14,0)	18 (8,1)	
**Lower vocational school**	23 (19,0)	27 (12,1)	
**Higher vocational school**	74 (61,2)	87 (39,0)	
**Higher education/University**	7 (5,8)	91 (40,8)	
**Nationality**	122	251	**0,002**
**Belgian**	105 (86,8)	240 (95,6)	
**Non-Belgian**	16 (13,2)	11 (4,4)	
**Subjective health perception**	120	237	**< 0,001**
**Good or very good**	73 (60,8)	203 (85,7)	
**Fair, bad or very bad**	47 (39,2)	34 (14,3)	
**Current smoker**	112	253	**< 0,001**
**Yes**	100 (89,3)	72 (30,5)	
**Contact with GP last 2 months**	120	238	**< 0,001**
**Yes**	77 (64,2)	104 (43,7)	
**Contact with secondary care last 2 months**	121	240	
**Yes**	38 (31,4)	44 (18,3)	**0,005**
**Contact with ED in last 2 months**	120	243	**< 0,001**
**Yes**	33 (27,5)	7 (2,9)	
**Type of GP practice**	75	224	**< 0,001**
**Single handed**	27 (36,0)	151 (67,4)	
**Two handed**	6 (8,0)	61 (27,2)	
**Group practice**	42 (56,0)	12 (5,4)	

* All significant results are indicated in bold.

Concerning health care utilisation, bivariate analysis demonstrate a significant association between being homeless and the likelihood to consult a GP (p < 0,001), secondary care (p = 0,005) and emergency care (p < 0,001), with higher likelihoods for all three forms of care in the homeless people.

Bivariate analysis also show an additional significant association between likelihood to consult a GP and perceived health status (p = 0,019). For the likelihood to consult an ED and likelihood to consult secondary care we found no significant associations (see table 2).

**Table 2:** health care use of the homeless people: descriptive and univariable results (total n homeless: 121)

	Had contact with GP	Total n		Had contact with ED	Total n		Had contact with secondary care	Total n	
	**n (%)**		P*	**n (%)**		P*	**n (%)**		P*

									
**Sex**			0,151		121	0,507		121	0,274
**Male**	63 (80,8)			29 (87,9)			30 (78,9)		
**Female**	15 (19.2)			4 (152,1)			8 (21,1)		
									
**Age**			0,693		120	0,466		121	0,171
**18-29**	24 (31,2)			12 (36,4)			12 (31,6)15 (39,5)		
**30-39**	20 (26,0)			11 (33,3)			7 (18,4)		
**40-49**	20 (26,0)			5 (15,2)			4 (10,5)		
**50-59**	11 (14,3)			4 (12,1)			0 (0,0)		
**60-65**	2 (2,6)			1 (30,0)					
**Educational level**			0,621		121	0,740		121	0,274
**No education/primary school**							3 (7,9)		
**lower education**	11 (14,1)			6 (18,2)					
**Lower vocational school**	15 (19,2)			7 (21,2)			9 (23,7)		
**Higher vocational school**	46 (59,0)			19 (57,6)			25 (65,8)		
**Higher education/University**	6 (7,7)			1 (3,0)			1 (2,6)		
**Nationality**			0,344		121	0,827		121	0,989
**Belgian**	66 (84,6)			29 (87,9)			33 (86,8)		
**Non-Belgian**	12 (15,4)			4 (12,1)			5 (13,2)		
**Subjective health perception**			**0,019**					121	0,116
**Good or very good**	41 (52,6)						19 (50,0)		
**Fair, bad or very bad**	37 (47,4)						19 (50,0)		
**Current smoker**			0,426		113	0,683		113	0,257
**Yes**	64 (87,7)			28 (87,5)			33 (94,3)		
**Type of GP practice**			0,396		76	0,647		76	0,629
**Single handed**	19 (31,7)			8 (44,4)			10 (40,0)		
**Two handed**	5 (8,3)			1 (5,6)			1 (4,0)		
**Group practice**	36 (60)			9 (50,0)			14 (56,0)		

When homeless people consult a GP, they do so at a group practice (56 % versus 5,4%), more often than when non-homeless patients consult a GP (see table 3).

**Table 3:** Type of practice

Type of practice	Homeless	Ghent sample
Solo	32,2%	64,6%
Duo	8,5%	28,3%
Group practice	59,3%	7,1%

To determine the independent impact of being homeless on the likelihood to consult a GP, secondary care and an ED, three multiple logistic regression models were built adjusting for sex and age (see table 4). The model focusing on likelihood to consult a GP explained 13,8% of the variance and demonstrated that even when adjusting for age and gender, homeless people have a significant higher likelihood to consult a GP (see table 4). The odds ratio for the homeless is 3,641 representing the homeless persons' increase in likelihood to consult a GP (95% CI = 2,160 - 6,139).

**Table 4:** Logistic regression model for going to the GP, the ED and secondary care in the past two months

Variable	Estimate	SE	Wald	P*	Odds ratio	95% CI
**Contact with GP**						

**Sex**	0,607	0,249	5,926	**0,015**	1,835	1,126-2,993
**Age**						
**18-29**			19,895	**0,001**		
**30-39**	-0,146	0,313	0,218	0,641	0,864	0,468-1,595
**40-49**	0,070	0,305	0,052	0,819	1,071	0,589-1,951
**50-59**	1,173	0,359	10,672	**0,001**	3,233	1,599-6,537
**60-65**	1,242	0,457	7,381	**0,007**	3,462	1,413-8,480
**Homeless in Ghent**	-1,292	0,266	23,523	**0,000**	3,641	2,160 -6,139

**Contact with emergency department**						

**Sex**	-0,032	0,475	0,005	0,946	0,969	0,382-2,456
**Age**						
**18-29**			5,929	0,205		
**30-39**	-0,107	0,450	0,057	0,811	0,898	0,372-2,168
**40-49**	-1,214	0,563	4,655	**0,031**	0,297	0,099-0,895
**50-59**	-0,743	0,621	1,434	0,231	0,476	0,141-1,605
**60-65**	0,149	0,866	0,030	0,863	1,161	0,213-6,339
**Homeless in Ghent**	-2,592	0,490	28,016	**0,000**	13,351	5,114-34,857

**Contact with secondary care**						

**Sex**	0,904	0,294	9,441	**0,002**	2,470	1,387-4,398
**Age**						
**18-29**			4,362	0,359		
**30-39**	0,391	0,359	1,187	0,276	1,478	0,732-2,986
**40-49**	-0,089	0,376	0,056	**0,813**	0,915	0,438-1,911
**50-59**	0,355	0,405	0,768	0,381	1,426	0,645-3,151
**60-65**	0,819	0,509	2,587	0,108	2,268	0,836-6,150
**Homeless in Ghent**	-2,592	0,305	15,130	**0,000**	3,271	1,800-5,943

* All significant results are indicated in bold.

Nagelkerke R² contact with GP: 0,138. Nagelkerke R² contact with ED: 0,277, Nagelkerke R² contact with secondary care: 0,087

The model focusing on likelihood to consult secondary care explained 8,7% of the variance and demonstrated after adjusting for age and gender, being homeless increases the likelihood to consult secondary care. The odds ratio for the homeless is 3,271 representing the homeless persons' increase in likelihood to consult secondary care (95% CI = 1,800 - 5,943).

The comparable model for likelihood to consult an ED explained 27,7% of the variance and demonstrates that being homeless also increases the likelihood to consult an ED, after adjusting for age and sex (see table 3). Homeless patients have a 13,351 times higher likelihood to consult an ED compared to the general population (OR: 13,351 95% CI = 5,114 - 34,857).

Regression models additionally adjusting for perceived health status follow the same trend. The explained variance increases to 21,5% for the model focussing on likelihood to consult a GP, to 11,9% for the model focussing on likelihood to consult secondary care and to 33,3% for the model focussing on likelihood to consult an ED. In these models, the OR for a homeless person to be likely to consult a GP is 2.592, to consult secondary care 2,683 and to consult an ED 10,752.

## Discussion and conclusion

Homeless people have a disproportionately higher burden of health problems than the general population. Existing studies (mainly from the UK and the US) describe higher utilisation rates for hospital-based care and emergency care, and lower rates for primary care by homeless people compared to the general population. Some studies indicate that homeless people do not access primary care at all and use emergency care as the only form of health care [6,16,20]. Homeless people are importantly hindered and/or steered in their health care use by barriers directly related to the organisation of care [6,7,21,24].

In this study we describe the likelihood to consult a GP, secondary care and emergency care of homeless people living in an area with a primary health care system in which homeless people with health problems are actively guided to low-threshold but universal accessible (i.e. not selective) community health centres.

Hereto the Belgian National Health Survey Database was extended with data of 122 homeless persons residing in one of the night shelters or temporarily accommodations for homeless people in Ghent.

The homeless respondents in this study are significantly younger, lower educated, more likely to smoke, have more often a non-Belgian nationality and report to be in worse health than the general population. These findings are concordant with other studies reporting that homeless people have worse health, suffer more from multimorbidity, have higher smoking prevalence (up to 80%), and are mostly less educated, decreasing the chance to find a stable job [4,6,14,18,31,32].

Literature shows a growing number of women in the homeless population [33]. This could not be confirmed in this study in which over 80% of the respondents are male. This could partly be due to a selection bias; women (often with children) who become homeless usually stay in crisis centres for women and children which were not included in this study. However, possibly there are also less homeless women in Belgium than in other countries because of the relatively good safety net for women with children; they have priority in getting social housing or -in case of the unavailability of social housing-emergency housing (temporarily housing in a premises owned or rented by the council).

Multivariate analyses show that homeless persons in Ghent have a higher likelihood to consult a GP (i.e. have consulted the GP at least once in the 2 months prior to the interview) than the general population after adjusting for sex, age and perceived health status. This finding indicates that in Ghent the homeless people find the way to primary health care and use it.

This contrasts with studies from other countries. Several US based studies show lower utilisation rates of ambulatory care and up to 5 times higher utilisation rates of acute hospital-based care and emergency care in the homeless population compared to the US average [6,16,20]. Studies from the United Kingdom describe the same trend: homeless people contact secondary services more frequently than the housed population. Some studies describe that a considerable proportion of the homeless population does not find the way to primary health care at all: a survey conducted by Crisis (the national charity for solitary homeless people in the UK) in 2002 reported that homeless people were three times more likely not to have had contact with a GP in the last year and were nearly five times more likely than the general population to attend the emergency departments [34].

A 2002 survey conducted in the UK also looked at the likelihood to consult a GP. They found that homeless persons were 3 times more likely not to contact their GP in the last year than the general population[34].

This higher likelihood in Ghent to consult a GP in the homeless population compared to the general population could be explained by the fact that primary health care for homeless people in Ghent is organised in a way that contributes to the reduction of some of the barriers demonstrated in others types of primary health care organisation such as financial barriers (insurance status), registration, accessibility, stigmatisation,... [7,11,14,21,23,24].

Tracing homeless people with health problems and active guidance towards primary care centres by social workers, might contribute to an easier access to the GP. The Community Health Centres are situated close the shelters and social restaurants, so transportation is often not an issue. There are also agreements on the payment procedure so the homeless people do not need to worry about money, reducing the financial barrier. Additionally, Community Health Centres are known not to stigmatise or refuse patients because of being homeless or having no money. This hypothesis is supported by the finding that the homeless people who visit a GP, do this considerably more often at a group practice (including the health centres) (56% versus 5,6% in the general population).

Also, the universal approach of the primary care centres (providing care to everyone residing in the geographical area) leads to less stigmatization and labelling than in a selective setting focussing solely on the homeless population. In this study, the higher likelihood to consult a GP does not go hand in hand with a lower likelihood to consult the emergency department or secondary care. This indicates that homeless people, although they have a higher likelihood to make use of the primary health care system, have not totally abandoned the way to emergency care. Probably this can partly be explained by the particularity of the health problems homeless people experience (e.g. postponing care until it's really urgent or care during the evening or night). Homeless people are also more often the victim of drug related accidents and road accidents and suffer from health problems that need more emergency care than the general population (e.g. hypothermia)[35]. Additionally, this study has a cross-sectional design which makes that we do not know whether the homeless people would use more frequently the emergency services if there was the universal primary care system would have been organised differently. Moreover, since this study is the first to study the health care use of homeless people in Ghent, it is not possible to describe an increase or decrease in the emergency care use of the homeless population over time.

This study has some limitations. First of all there is no reliable information available on the response rate of the homeless people nor on the respondents excluded from the study. The study was presented to all homeless persons present at different moments in one of the shelters in Ghent during the five months inclusion period and everyone was invited when fulfilling the inclusion criteria. However, there was no registration of any information concerning the people who did not want to participate. This was mainly due to anonymity reasons thrown up by the managers of the shelters. Also the exact total number of homeless people visiting shelters and homeless centres is not available. Most of the crisis centres have no registration of their visitors.

A second and important limitation of this study is that only likelihood to consult is studied. The National Health Survey contains self reported frequencies of health care use, so analogously these frequencies were also included in the survey for the homeless people. However, most of the respondents found it very hard to remember the exact number of their visits to a care provider. For a more nuanced view on health care use, information on the number of consultations is necessary. The concept of likelihood to consult is in any case usable to evaluate whether people know and find their way to a certain care provider but not to evaluate the intensity of their health care use. Although this study cannot prove the impact of a better accessible primary health care system on emergency care utilisation rates or on secondary care utilisation rates, a recent study commissioned by the Belgian government gives an important indicator of the possible impact. In this report the authors compared the traditional, fee-for-service based primary health care services in Belgium with capitation based primary health care services such as the community health centres. Their findings complement the findings of our study as they state that the community health centres indeed succeed in reaching the most vulnerable of society. Additionally this study provides evidence that people who found their way to these centres tend to contact less frequently the emergency department or secondary care[36].

The merits of this study lie in the fact that a relatively large sample of homeless people was questioned (an estimated third of the homeless population in Ghent visiting a homeless centre in the research period), following the research protocol and instruments of the National Health Survey as close as possible so that a comparison with National Health Survey data was made possible. However, prudence is recommended in the generalisation of the results. Data were collected from homeless people seeking help or assistance, mainly for food or a place to sleep, in the homeless centres or night shelters in Ghent. Without doubt their help seeking behaviour towards health care is different from that of homeless people not seeking help in the homeless centres. Also no information is available on the response rate and the characteristics of the people who refused to participate in the study, which makes it very difficult to evaluate the selection bias.

Further research addressing the health care use of the homeless people living in Belgium is needed to provide policymakers with the evidence needed to take action in order to make the health care system accessible and suitable for every person disregarding their different socio-economic backgrounds. The performance of universal primary health care services, as demonstrated in this study, may stimulate the debate between a universal or a selective approach.

## Competing interests

The authors declare that they have no competing interests.

## Authors' contributions

MVDW, TV, BA, SW and JDM contributed to the design of the study. MVDW and TV contributed to the data retrieval. EV and SW contributed to the analysis and the writing of the paper. BA, SW and JDM participated in the revision of the manuscript. All authors have read and approved the final manuscript.

## Pre-publication history

The pre-publication history for this paper can be accessed here:

http://www.biomedcentral.com/1472-6963/10/242/prepub
